# Understanding barriers to rehabilitation: child and family determinants of service utilisation in the Enabling Inclusion programme in rural South India

**DOI:** 10.1136/bmjpo-2025-003663

**Published:** 2026-03-31

**Authors:** Franzina Coutinho, Gauri Saxena, Marie Brien, Deepalaxmi Poojari, Selvam Ramachandran, Nisha Bhrasadiya, Sankara Raman Srinivasan, Navamani Venkatachalapathy, Noemi Dahan-Oliel, Dinesh Krishna

**Affiliations:** 1 Amar Seva Sangam, Aiykudi, Tamil Nadu, India; 2 Inspirium Holistic Care, Mumbai, Maharashtra, India; 3 McGill University, Montreal, QC, Canada; 4 University of Toronto, Toronto, Ontario, Canada; 5 Manipal Academy of Higher Education, Manipal, Karnataka, India; 6 Shriners Hospital for Children, Montreal, QC, Canada

**Keywords:** Rehabilitation, Health services research, Noncommunicable Diseases, Technology

## Abstract

**Background:**

Early identification and consistent intervention are essential to optimising developmental outcomes in children with neurodevelopmental disabilities. However, evidence on service utilisation patterns in low-resource contexts remains limited. This study examined the demographic characteristics and rehabilitation service utilisation of children enrolled in an early intervention programme, called the Enabling Inclusion (EI) programme in rural South India.

**Methods:**

A retrospective analysis of 6069 children receiving EI services between 2015 and 2024 was conducted. The investigators set a threshold for adequate therapy utilisation as children having received at least 42 therapy sessions over a 6-month period. Descriptive statistics and inferential analyses (including analysis of variance and Kruskal-Wallis tests) were used to explore the relationships between demographic factors (age, sex and diagnosis) and service utilisation outcomes, including number of therapy visits and duration of programme participation.

**Results:**

The average age at enrolment was 8 years and 7 months, with a male predominance (58%). Cerebral palsy (25%) and speech and language disorders (16%) were the most common diagnoses. Children under 6 received significantly more specialist visits (age: β=–5.19, p<0.001; age²: β=0.38, p<0.001), while older children relied more heavily on community rehabilitation workers (age: β=–11.30, p<0.001; age²: β=0.725, p<0.001). Children with intellectual disability, autism and cerebral palsy were significantly more likely to meet the threshold for adequate therapy utilisation, while those with hearing impairments and specific learning disabilities showed lower service uptake (p=0.034).

**Conclusion:**

The findings highlight age-specific and diagnosis-specific patterns in rehabilitation use, underscoring the need for adaptable, community-based strategies that ensure equitable access to early intervention in rural, resource-limited settings.

**WHAT IS ALREADY KNOWN ON THIS TOPIC**

**WHAT THIS STUDY ADDS**

- This study describes the age of diagnosis and enrolment to an early intervention programme, demographic characteristics and service utilisation patterns of children with neurodevelopmental disabilities in rural India.

**HOW THIS STUDY MIGHT AFFECT RESEARCH, PRACTICE OR POLICY**

- The findings of this study indicate that there is a pressing need to focus on early identification and screening services for children at risk of neurodevelopmental disabilities. Models of care using technology and empowering community rehabilitation workers are presented and can be scaled to increase the scope of rehabilitation service delivery, particularly to low-income families.

## Introduction

 Rehabilitation has been recognised as an essential component of health systems globally. In 2017, the World Health Organization’s Rehabilitation 2030: A Call for Action initiative emphasised that strengthening rehabilitation services is necessary for achieving the Sustainable Development Goals, including ensuring health and well-being for all people.^1^ Among the priority areas identified were the development of comprehensive rehabilitation delivery models and the collection of high-quality, population-level data to inform evidence-based planning.^1^ Robust health information systems are therefore crucial. At the clinical level, they guide decision-making and service provision, and at the policy level, they shape investment, workforce planning and equitable programme design.^2^

Neurodevelopmental disorders (NDDs) are a diverse group of conditions that affect cognitive, motor, communication and adaptive functioning from early childhood through the lifespan.^3^ Their aetiology is influenced by genetic, environmental and perinatal factors.^4 5^ Early detection and intervention substantially enhance developmental outcomes, reduce caregiver strain and support school and community participation.^6 7^ However, global prevalence estimates vary widely from 4.7 to 8.5 per cent due to methodological and sociocultural differences across regions.^3^ Evidence from India suggests an even higher burden, with estimates up to 13.6 per cent among children aged 2 to 9 years.^8^

Despite this high need, many low- and middle-income countries, including India, lack comprehensive, region-specific rehabilitation data.^9 10^ The absence of population-level utilisation data limits the ability of lower-middle-income country (LMIC) health systems to evaluate unmet rehabilitation needs, track equity gaps and plan scalable interventions, an area of increasing relevance to global rehabilitation policy.^10^ Challenges such as population diversity, fragmented health systems, sociocultural barriers and limited infrastructure hinder systematic data collection and restrict the ability to produce generalisable estimates.^11^ The absence of reliable information on service access, utilisation patterns and demographic disparities complicates policy decisions and limits the development of scalable and equitable rehabilitation models.^10^

India has implemented several initiatives, including District Early Intervention Centres and digital innovations in community-based rehabilitation. District Early Intervention Centres (DEICs) are government-run and provide multidisciplinary screening, therapy and referral services for children with developmental delays.^12^ Several non-governmental organisations (NGOs) complement these efforts with ground-level work to reach underserved and rural populations. For example, Equip India Charitable Trust trains and deploys Village Rehabilitation Workers for rural outreach and rehabilitation camps.^13^ Jan Vikas Samiti has a tele-rehabilitation initiative called Project Sambhav which uses a smartphone or web portal to connect rural CBR workers with specialists and deliver rehab services to children across several states.^14^ Samarthanam Trust for the Disabled works broadly on education, vocational training and rehabilitation services for persons with disabilities across India.^15^ These governmental and non-governmental initiatives work in tandem to strengthen India’s rehabilitation ecosystem.

However, large, population-based datasets describing real-world rehabilitation use remain scarce.^16^ Previous studies on the Enabling Inclusion (EI) model and programme in South India have demonstrated improvements in caregiver outcomes, child functioning and monitoring, evaluation and learning systems.^6 17 18^ However, these studies have focused on feasibility, short-term outcomes, or single-condition cohorts and have not examined long-term patterns of service utilisation across diagnostic groups, age ranges or socioeconomic gradients. This represents a critical evidence gap for understanding real-world engagement at scale.

Very little is known about how families in rural South India engage with community-based early intervention programmes, how demographic factors shape therapy uptake, or how blended models of specialist and community worker delivery function over many years. Large and region-specific datasets describing rehabilitation use in rural low- and middle-income settings are rare, and most available studies rely on small samples, facility-based recruitment or short follow-up periods.

The present study aims to address these gaps by analysing a decade-long dataset of 6069 children enrolled in Amar Seva Sangam’s EI programme. By examining demographic characteristics, diagnostic patterns and predictors of adequate service utilisation, this study provides one of the most comprehensive programme-based descriptions of early intervention engagement available in the Indian context. These findings can inform scalable, equitable and contextually responsive rehabilitation models for children with neurodevelopmental disabilities in low-resource settings.

## Methods

### Description of ASSA’s EI programme

Amar Seva Sangam (ASSA) is a non-governmental organisation that provides services to assist children and adults with disabilities in rural India. Their early intervention and child rehabilitation programme, called EI, is a community-based early intervention model supported by a digital platform (the EI App). It enables screening, assessment, individualised goal setting and therapy delivery by rehabilitation specialists and trained community rehabilitation workers (CRWs). The programme follows a structured, hybrid model consistent with the EI framework described in previous manuscripts.^6^,^8^ CRWs conduct developmental screening within rural communities using standardised tools embedded in the EI App. Children who screen positive are flagged within the platform and referred to a specialist for an evaluation.

Rehabilitation professionals (occupational therapists, physiotherapists, speech and language therapists or special educators) conduct detailed assessments and, in collaboration with families, develop individualised intervention plans. The initial phase typically includes 8–12 clinic-based sessions delivered by the rehabilitation specialist over approximately 2 months to establish goals, provide caregiver education and model therapeutic strategies.

Following this initiation phase, ongoing intervention is delivered primarily by the CRW within the child’s home or community setting at least once a week, guided by goals documented in the EI App. All visits and progress notes are recorded within the digital platform to enable monitoring and continuity of care. The rehabilitation specialists conduct at least one follow-up session per month to monitor progress, with additional sessions provided as needed based on clinical judgement.

Formal reassessment is conducted at least once every 6 months by the rehabilitation specialist, either through in-person visits or teleconsultation. Goals are reviewed in collaboration with the family, and modifications are documented in the platform. Updated plans are communicated to the CRW for continued implementation.

Within this model, rehabilitation specialist visits refer to sessions delivered by occupational therapists, physiotherapists, speech-language therapists and special educators, as documented in the EI App. Physiotherapy addresses gross motor development, mobility training, postural management and motor learning. Occupational therapy focuses on fine motor skills, functional participation, sensory processing and adaptive skills. Speech and language therapy includes receptive and expressive language intervention, augmentative communication and feeding support. Special education services address foundational academic readiness and cognitive development.

The programme is designed to improve access to early intervention and rehabilitation services for children with disabilities in rural and underserved areas. Previous evaluations of the EI approach have reported improvements in child developmental outcomes and caregiver-related measures.^6^,^817 18^ As of 2024, ASSA has provided services to more than 6000 children through this programme.

#### Study design and setting

This observational study involved retrospective analysis of programme data from the EI programme, implemented by Amar Seva Sangam (ASSA) between 2015 and 2024. The programme operates in three districts in the southern Indian state of Tamil Nadu—Tenkasi (population: 1 440 785), Tirunelveli (1 982 000) and Tuticorin (1 750 176).^19^

#### Participants

All children enrolled in the EI programme during the study period were included. Inclusion criteria required that children had not previously received regular early intervention or rehabilitation services at the time of enrolment. This was done to reduce confounding by variables such as initial skill level, rate of progress and utilisation patterns, which would make it harder to isolate the relationship between child/family factors and utilisation within the EI programme itself. In addition, the programme was geared towards enrolment of children who did not have access to any other regular early intervention services through other public, private or non-profit sources. Children were grouped into 2-year age intervals ranging from 0 to 1 years to 18 years. The study was approved by the Institutional Ethics Committee at Kasturba Medical College and Kasturba Hospital, Manipal Academy of Higher Education, India (IEC2: 393/2023). Participants were not involved in the design, conduct of research or dissemination of this research.

### Adequate Service Utilization

Adequate service utilisation was defined as a dichotomous outcome variable (yes/no), indicating whether a child attended at least 42 therapy sessions over a 6-month period. This threshold was established using a data-driven approach incorporating median session attendance, percentile-based distributions and observed engagement patterns across the cohort. The underlying measure—total therapy sessions attended—was initially treated as a continuous variable, then categorised using a binary cut-off, where children with ≥42 sessions were labelled ‘adequate utilizers’ and those with <42 sessions as ‘low utilizers’. This threshold reflects programme expectations (eg, weekly therapy over 6 months) and was designed to balance statistical robustness with clinical relevance. It provides an objective indicator of service receipt over time, independent of prescribed dosage or caregiver adherence, and supports standardised comparisons across demographic and diagnostic groups.

### Sociodemographic Variables and Measurement

Key predictor variables were collected at enrolment through caregiver interviews conducted by trained staff and recorded within the EI App.

Age at enrolment was calculated in months based on the child’s date of birth and the date of entry into the programme and used as a continuous variable in all analyses.

Maternal education was captured through a closed-ended question with predefined categories: (1) no formal education, (2) Anganwadi/pre-school, (3) 1st–12th standard (elementary to higher secondary), (4) diploma, (5) undergraduate degree and (6) postgraduate degree. For statistical modelling, maternal education was analysed as a categorical variable and was also dichotomised (≥secondary education vs.<secondary education) in select analyses.

Household income was self-reported via a closed-ended question and categorised into four income groups based on Indian rupee (INR) thresholds aligned with World Bank country classifications: (1) low income (<₹125 000 /year), (2) lower-middle income (₹125 000–500 000/year), (3) upper-middle income (₹500 000–3 000 000/year) and (4) high income (>₹3 000 000/year). For some models, income was dichotomised (low income vs all others) to assess the impact of socioeconomic status on service utilisation.

These variables were selected a priori based on empirical evidence and theoretical frameworks highlighting their role as social determinants of health. They were included as covariates in multivariable regression models to evaluate their independent association with the likelihood of achieving adequate rehabilitation service utilisation.

### Statistical analysis

To describe the demographic characteristics and rehabilitation service utilisation patterns of children enrolled in the EI programme, descriptive statistics were used. Data was analysed to report descriptive statistics including age at enrolment, the average days in the EI programme and the number of rehabilitation specialist and CRW visits for each age group, stratified by sex. To examine the associations between demographic variables and the adequacy of service utilisation, linear regression models were conducted to explore the association between age, sex and the number of specialist and CRW visits, with a particular focus on identifying patterns in service utilisation. SPSS V. 29.0 (IBM Corp., Armonk, New York, USA) was used for all descriptive and analytical statistics, with a p value of less than 0.05 set for statistical significance.

Data completeness and quality were reviewed prior to analysis. All analyses were conducted on the most complete and verified dataset available, and systematic checks were performed to increase internal validity. Records with critical missing fields, such as date of enrolment, diagnosis or therapy session counts, were excluded from regression analyses but retained for descriptive summaries where applicable. No imputation was performed for missing data. Manual and automated consistency checks were applied across variables to reduce entry errors, and duplicate entries were removed. The dataset used represents the most complete and valid records available from ASSA’s EI programme database during the 2015–2024 period. The dataset was initially developed for operational monitoring rather than research purposes. This introduced structural constraints on variable completeness, particularly for early programme years where discipline-specific therapy counts and certain demographic indicators were inconsistently documented.

## Results

During the study period (2015–2024), a total of 6069 children were diagnosed with NDDs and enrolled in the EI programme at ASSA.

### Demographic information

#### Age of enrolment and rehabilitation

Children enrolled in ASSA’s Early Intervention (EI) programme ranged in age from 0 to below 19 years. The average age at enrolment was 8 years and 7 months, with a median of 8 years and 4 months (SD=4.5). The highest enrolment was observed among children aged 7 to 8 years (10.1%, n=615), whereas the lowest enrolment occurred in the 0–1 year age group (0.6%, n=37). With respect to sex, the cohort was predominantly male (58%) (online supplemental table 1).

#### Demographic factors and service utilisation

Service utilisation was operationalised as the total number of therapy sessions attended over a 6-month period.

**Figure 1:**
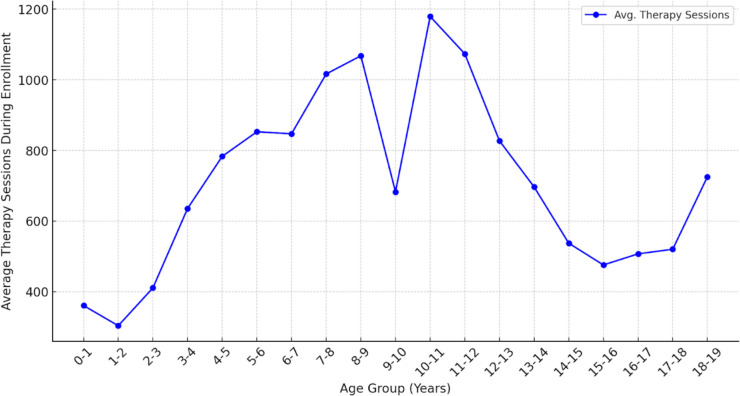
Mean number of therapy sessions by age at enrolment.

To examine whether age at enrolment was associated with service utilisation, Spearman’s rank-order correlation was conducted, which revealed a statistically significant but weak negative correlation between age at enrolment and total therapy sessions attended (ρ=–0.22, p=0.019). This correlation was not significant after adjusting for sociodemographic variables (AOR=0.98 per month increase in age; 95% CI 0.94 to 1.02; p=0.26). In contrast, higher maternal education and household income were significantly associated with greater service utilisation. Children whose mothers had at least a secondary school education were more likely to achieve adequate service utilisation (χ²(1) = 7.89, p=0.005), and similar trends were observed among children from higher-income households. In addition, a one-way analysis of variance (ANOVA) was conducted to explore whether time spent in the EI programme differed by sex. The analysis revealed a statistically significant difference in average days spent in the programme between males and females (F(1, 34) = 6.10, p=0.018), with males spending more days in the programme on average than females (figures1  2). This analysis was based on aggregated age-group data.

**Figure 2:**
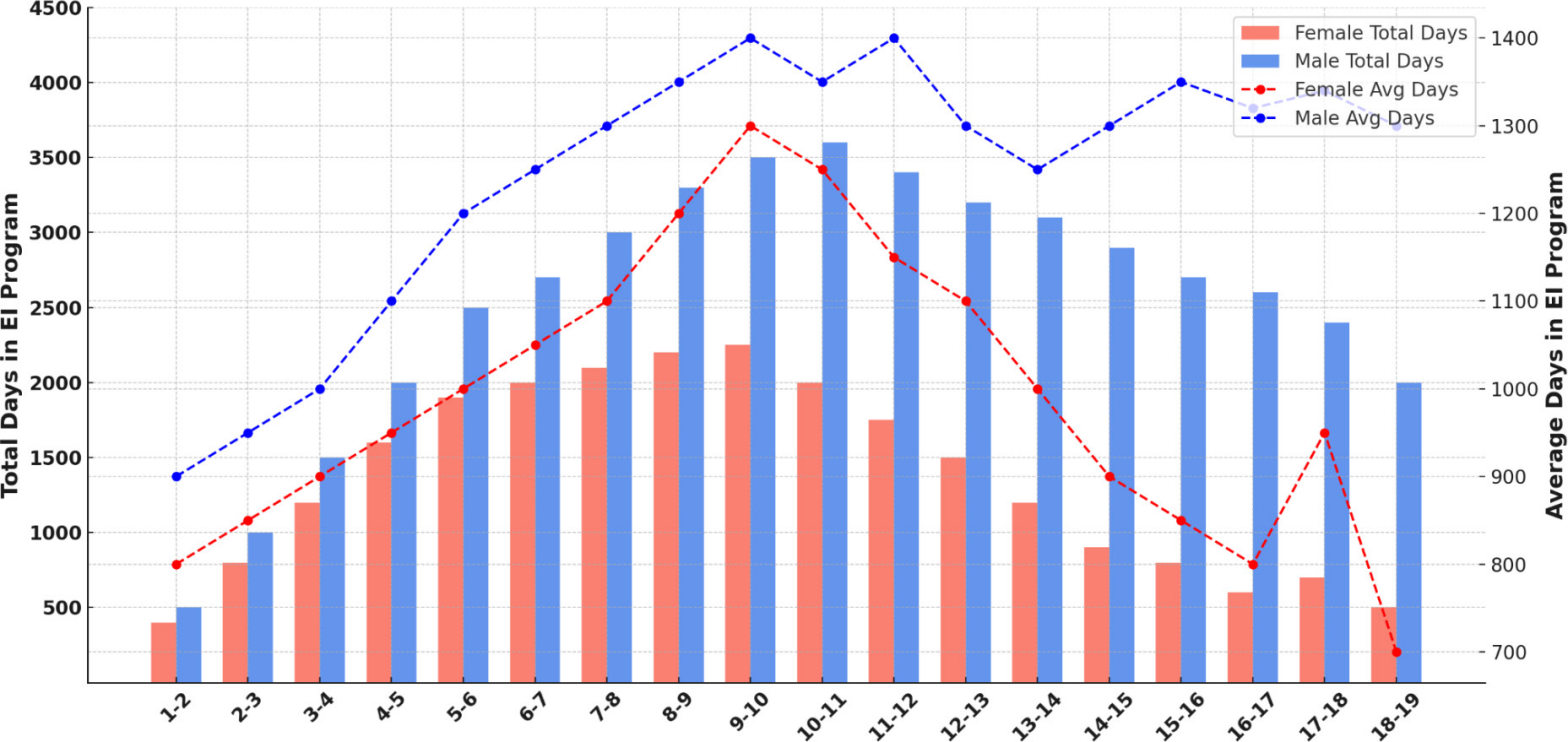
Average number of days spent in the EI programme by age and sex.

#### Prevalence and service utilisation based on diagnosis

The most common diagnosis was cerebral palsy (25%, n=1546), followed by speech and language disorder (16%, n=1022) (*‘Speech Language Disorder’ was recorded as a standalone diagnosis in the dataset in cases where the child’s primary presenting difficulty was related to speech and/or language, and where no other neurodevelopmental intellectual disability diagnosis had been made at the time of enrolment*), hearing impairments (14%, n=869) and autism (7.5%, n=769).

#### Statistical relevance of the threshold

The median-based approach for adequate utilisation ensures that half of the children attended 42 or more sessions, while the other half attended fewer, providing an empirical and non-arbitrary benchmark for defining adequate utilisation. Based on this classification, 51.6% (n=3134) of children were categorised as ‘adequate utilizers’, while 48.4% (n=2935) were classified as ‘low utilizers’.

A Kruskal-Wallis test (p=0.034) demonstrated significant differences in service utilisation across diagnostic categories. Post hoc comparisons indicated that children with intellectual disability (82.2%, n=714), autism (79.6%, n=612) and cerebral palsy (76.6%, n=1185) were significantly more likely to achieve adequate utilisation compared with those with specific learning disabilities (52.5%, n=160) and hearing impairments (49.7%, n=146).

#### Service utilisation and time spent in the programme based on diagnosis

Children with a diagnosis of intellectual disability had the highest number of average visits (n=115.9), followed by Down syndrome, ADHD and cerebral palsy. Children with autism and speech and language disorders showed lower average utilisation.

An ANOVA was conducted to assess the differences in the average number of service days spent in EI programmes across various diagnostic categories. Significant group differences were observed in EI service visits across diagnoses (F(5, 5803) = 1345.95, p<0.001), indicating that at least one diagnosis differed significantly from the others. Specifically, intellectual disability (115.9 visits), Down syndrome (91.9 visits) and CP (85.1 visits) were associated with a higher average number of EI service visits compared with speech and language disorder (73.6 visits).

**Figure 3:**
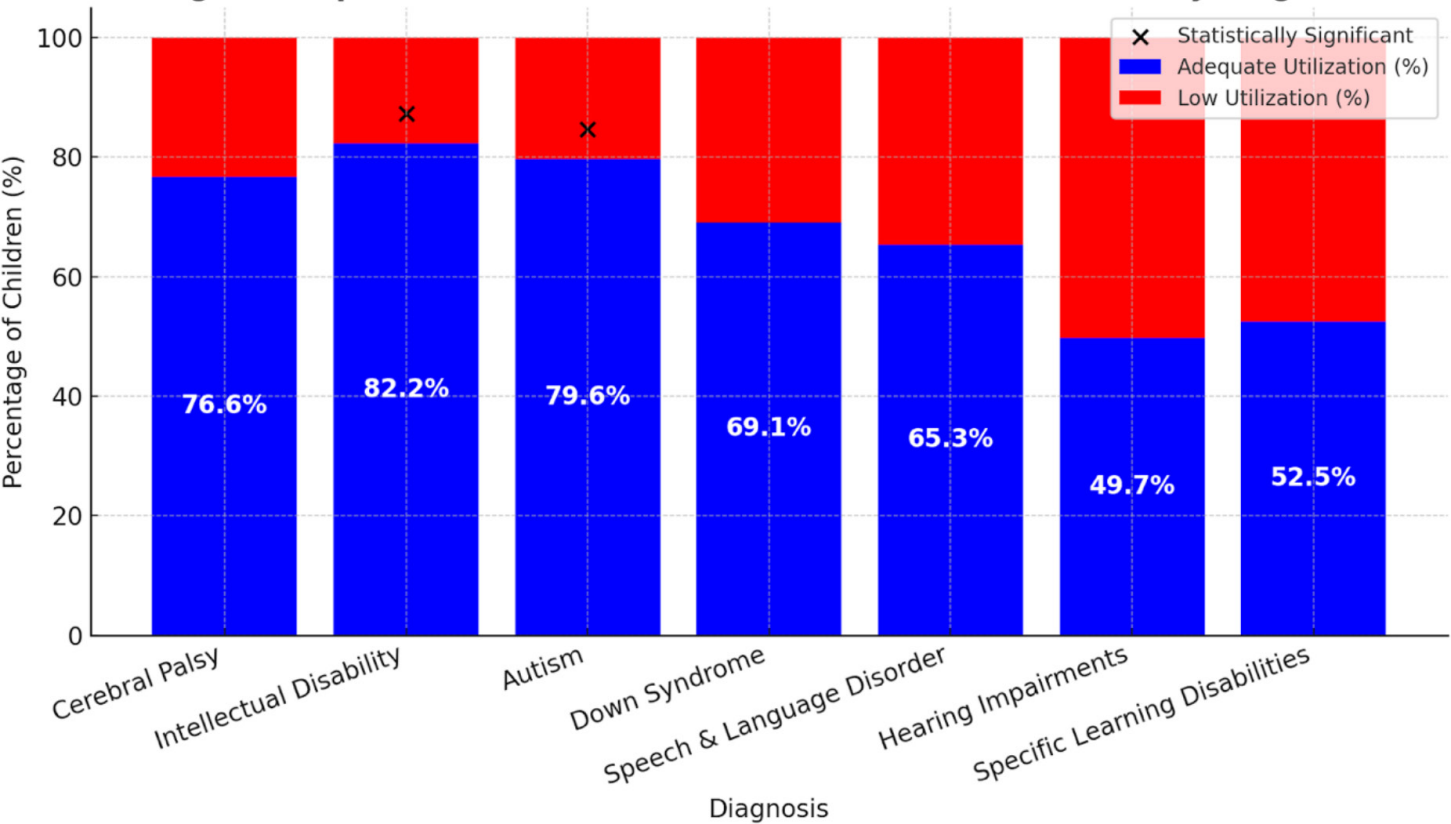
Adequate versus low utilisation of rehabilitation service by diagnosis. The statistical significance note is positioned on the side, specifying that significant differences were observed in intellectual disability and autism.

To further explore these differences, Tukey’s honest significant difference (HSD) test was performed as a post hoc analysis. The test confirmed that the average number of EI service visits for intellectual disability, Down syndrome and CP was significantly higher than for speech and language disorder, with mean differences of 30.8, 6.8 and 11.5 visits per child, respectively. No statistically significant differences were observed between other diagnosis pairs, such as ADHD (89.8 visits) and autism (65.5 visits), indicating similar service utilisation patterns (table 1 and figure 3).

**Table 1:** Diagnoses and rehabilitation visits at ASSA via the Enabling Inclusion program**®** from 2015 to 2024

Diagnosis	Number of children	Specialist visits (total; mean; min–max)	CRW visits (total; mean; min–max)	Total visits
Cerebral palsy	1546	67 138 (43.4; 39–650)	64 477 (41.7; 32–987)	131 615
Intellectual disability	869	61 875 (71.2; 65–780)	38 838 (44.7; 19–828)	100 713
Speech and language disorder	1022	31 536 (30.9; 26–365)	43 727 (42.8; 20–465)	75 263
Autism	769	30 336 (64.7; 56–684)	20 018 (42.7; 33–984)	50 354
Down syndrome	219	10 739 (49.0; 26–800)	9394 (42.9; 10–900)	20 133
ADHD	188	9327 (49.6; 42–1062)	7549 (40.1; 10–1162)	16 876
Hearing impairment	294	7056 (24.0; 20–478)	9451 (32.1; 10–500)	16 507
Multiple disability	172	6629 (38.5; 30–463)	6885 (40.0; 38–526)	13 514
Specific learning disability	305	5157 (16.9; 12–276)	6432 (21.1; 18–276)	11 589
Delayed developmental milestones	161	3135 (19.5; 10–451)	4602 (28.6; 12–561)	7737
Locomotor disability	85	1641 (19.3; 18–215)	2891 (34.0; 20–198)	4532
Spina bifida	48	1079 (22.5; 16–280)	1607 (33.5; 16–256)	2686
Global developmental delay	25	683 (27.3; 20–165)	801 (32.0; 20–185)	1484
Blindness	23	411 (17.9; 10–309)	579 (25.2; 10–379)	990
Other	343	2228 (6.5; 3–406)	4790 (14.0; 11–196)	7018

Values represent total number of visits and mean visits per child within each diagnostic category. Ranges indicate minimum and maximum visits per child.

ASSA, Amar Seva Sangam; CRW, community rehabilitation worker.

Rehabilitation visits according to age and sex (online supplemental table 2).

### Specialist visits

Our analysis of rehabilitation specialist service provision across different age groups and sex reveals distinct patterns in healthcare utilisation. In the youngest age group (0–1 years), females average 61.79 visits compared with 53.61 visits for males (SD 14.25). In older children, the trend reverses with more utilisation for males. For example, in the 4–5 years age group, females average 30.61 visits (SD 115.25), while males average 43.13 visits (SD 141.75). This pattern persists across older age groups.

A quadratic regression model incorporating both age and sex provided the best fit for predicting the nonlinear pattern of utilisation of specialist visits. The analysis demonstrated a nonlinear (U-shaped) relationship between age and the number of specialist visits, where utilisation declines in early childhood but increases again during later years. The coefficient for age was negative (β=–5.1855, p<0.001), indicating that specialist visits initially decrease with age. However, the age² coefficient was positive (β=0.3808, p<0.001), confirming an upward trend in specialist visits as age progresses. The minimum point of specialist utilisation occurred at approximately 6.8 years of age, after which utilisation began to rise again for both girls and boys. Furthermore, males received approximately 7.10 more visits than females (β=7.0972, p<0.001), suggesting that sex plays a significant role in specialist service utilisation. This model explained 39.1% of the variance in specialist visits.

### Community rehabilitation worker visits

**Figure 4:**
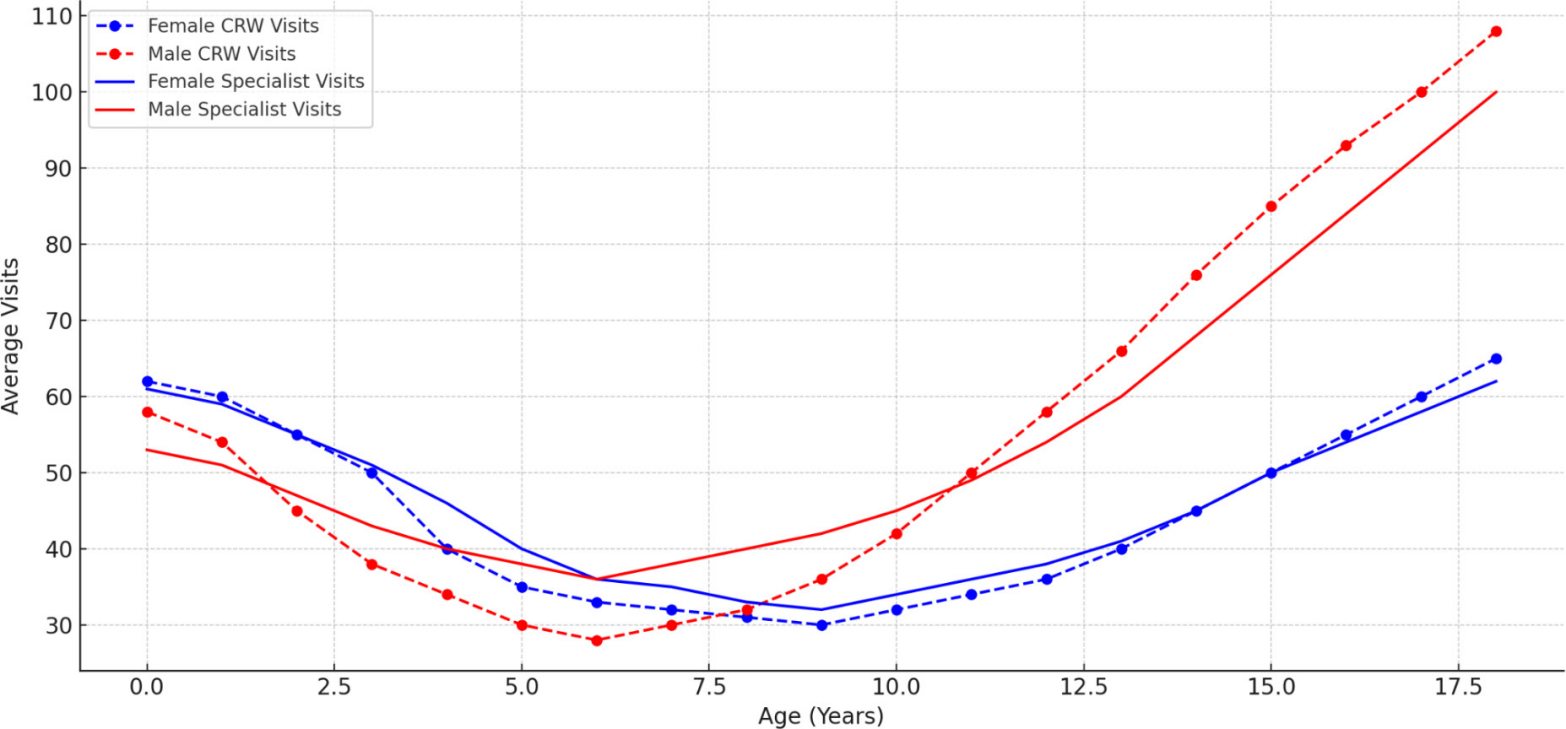
CRW versus specialist visits by age and gender.

The descriptive statistics for CRW visits reveal distinct patterns concerning age, sex and the number of visits. Analysis shows that CRW visits followed a non-linear age-dependent trend, with younger children receiving more frequent visits. In the 0–1 age group, females averaged 86.3 visits (SD: 16), while males averaged 93.7 visits (SD: 17.75). In the 5–6 age group, females received an average of 40.4 visits (SD: 99), while males averaged 43.3 visits (SD: 107.25). By adolescence (15–16 age group), the frequency decreased significantly, with females averaging 30.0 visits (SD: 94.5) and males 52.6 visits (SD: 129.25). Interestingly, while the frequency reduced with age, males often received slightly more visits in the middle and older age groups compared with females. Polynomial regression analysis was conducted to examine the association of age and sex on the number of CRW visits. The model revealed that age significantly impacts CRW service provision in a non-linear manner, visits initially decline with age (β=−11.30, p<0.001), but later increase again in adolescence (β=0.725, p<0.001). This suggests that CRW visits follow a U-shaped trajectory, rather than a simple linear increase or decrease (figure 4).

#### Parental education level

A χ^2^ test for independence was conducted to examine the differences in education levels between fathers and mothers. The test revealed a statistically significant difference (χ² (5, n=6069) = 182.73, p<0.001), indicating that educational attainment distributions differ between sexes in this sample.

Children whose mothers had at least a secondary school education were significantly more likely to meet the adequate utilisation threshold (82%, n=2568), compared with only 50% (n=280) of children whose mothers had no formal schooling (χ²(1) = 7.89, p=0.005) (table 2).

**Table 2:** Parental education level

Education level	Fathers (n)	Mothers (n)
No formal education	452 (7.4%)	560 (9.2%)
Anganwadi (pre-school)	53 (0.9%)	46 (0.8%)
1st–12th standard	4430 (73%)	4176 (68.8%)
Diploma	449 (7.4%)	314 (5.2%)
Undergraduate	470 (7.7%)	633 (10.4%)
Postgraduate	215 (3.5%)	340 (5.6%)
Total	6069	6069

### Income levels

Close to 90% of families in this study fell into the low-income category, with an annual household income of less than ₹125,000, aligning with the World Bank’s low-income country (LIC) classification (GNI per capita <US$1135) (n=5413, 89.2%). A smaller proportion (n=590, 9.4%) belonged to the lower middle class, earning between ₹125 000 and ₹500 000 annually, a range overlapping with the World Bank’s lower-middle-income countries (LMICs) (GNI per capita US$1136–US$4465). Only 1.0% (n=64) of children were from upper-middle-class households earning ₹500 000–₹3 000 000 annually, corresponding to upper-middle-income countries (UMICs) (GNI per capita US$4466–US$13 845). A negligible proportion (n=2, 0.03%) belonged to high-income households with earnings above ₹3 000 000 annually, aligning with the high-income country (HIC) classification (GNI per capita >US$13 846) (table 3).

**Table 3:** Annual parent income levels

Income level	Gross annual income (Rs. ×1000)	No. of children (n)	Percentage (%)	Approximate world bank classification
Low income	<125	5413	89.2%	Likely LIC (low income, <US$1135 GNI per capita)
Lower middle class	125–500	590	9.4%	Overlaps LIC and LMIC (US$1136–US$4465 GNI per capita)
Upper middle class	500–3000	64	1.0%	Overlaps LMIC and UMIC (US$4466–US$13 845 GNI per capita)
High income	>3000	2	0.0%	Likely HIC (high-income, >US$13 846 GNI per capita)
Total	–	6069		–

### Perinatal and postnatal risk factors for children

Perinatal and postnatal risk factors for NDDs are highlighted in table 4.

**Table 4:** Perinatal and postnatal status for children who were born with NDD in the ASSA EI programme

Perinatal status	No. of children (n)	Percentage (%)
Perinatal status		
Delayed birth cry or no birth cry	591	8.2
Premature baby	227	3.2
Forceps delivery	140	1.9
Birth asphyxia	129	1.8
Prolonged labour	119	1.7
Neonatal convulsions	60	0.8
Abnormal colour	52	0.7
Breech presentation	41	0.6
Cord around the neck	39	0.5
Hydrocephalus	34	0.5
Home Delivery	32	0.4
Microcephaly	29	0.4
Postnatal status		
No issues	3393	48.6
Meningitis	56	0.8
Convulsions	319	4.6
Jaundice	219	3.1
Trauma/accident	27	0.4
General development delay	1101	15.8
Low birth weight	963	13.8
High birth weight	107	1.5
Infections	140	2.0
Cardiac abnormalities	49	0.7
Respiratory difficulties	189	2.7
Other	414	5.9

ASSA, Amar Seva Sangam; EI, Enabling Inclusion; NDD, neurodevelopmental disability.

The data indicate that the majority of children (n=5582; 92.0%) did not have high-risk perinatal features. Among postnatal high-risk indicators, 1101 children (15.8%) had general developmental delay, 963 (13.8%) had low birth weight and 319 (4.6%) experienced convulsions. Perinatal risk factors were not mutually exclusive, and children may have had more than one reported risk factor.

## Discussion

This study described the demographic characteristics and rehabilitation service utilisation pattern of children with NDDs in rural South India. The study explores region-specific patterns by focusing on three districts where an NGO-led rehabilitation programme served children without prior access to formal early intervention. By generating detailed, region-specific utilisation trends, this study responds directly to ongoing calls to strengthen needs assessment rigour for paediatric rehabilitation in LMIC contexts. Our findings offer insights into the sociodemographic factors associated with therapy engagement in South India within the constraints of this programme-based data set.

### Age of enrolment and rehabilitation

Globally, the median age for NDD diagnosis is around 4 years, but delays persist in LMICs where diagnosis often occurs after age 5–7.^20^ In this study, most children were enrolled between 6 and 10 years, suggesting delayed access compared with global benchmarks.^21^ However, age at enrolment was not significantly associated with continued therapy engagement after accounting for other sociodemographic factors measured in this study. Instead, parental education and socioeconomic status had stronger influences. These findings align with prior research linking delayed diagnosis and low service uptake to socioeconomic disparities and awareness gaps.^11 22^ Rural infrastructure, stigma and cultural beliefs are likely to contribute to these delays.

Expanding community-based screening, parental education and stronger referral pathways can facilitate earlier identification and access to services during critical developmental periods. This is especially relevant for younger children and girls who are often underrepresented in early intervention services.

### Prevalence and service utilisation by diagnosis

Children with intellectual disability, autism and cerebral palsy showed the highest rates of adequate utilisation, likely due to their complex and multi-domain needs requiring intensive therapy.^7 23^ Conversely, children with specific learning disabilities and hearing impairments had lower utilisation, possibly due to differing models of care or lower perceived urgency.^24^ These disparities underscore the importance of tailoring service intensity and models to diagnostic categories.

When considering time in the programme, service intensity did not always correspond with visit counts. For example, children with Down syndrome had more frequent, shorter interventions, while those with CP required fewer but longer sessions. These findings highlight the need for diagnosis-specific care planning. Previous research on community-based rehabilitation and early intervention in India has largely focused on clinical outcomes, caregiver experiences or single condition cohorts. Far less is known about population level service utilisation trends, especially across diverse diagnostic groups and within a digitally supported, community delivered model such as the EI programme.

### Predictive insights for service planning

CRW and specialist visits were stronger predictors of overall therapy attendance than diagnosis. This reflects both greater need and inequities in access, although clinical severity could not be directly examined in this dataset.^25^ Our binary, evidence-based definition of ‘adequate utilisation’ provides a practical metric to evaluate programme effectiveness.

The high correlation between CRW and specialist visits (r=0.94) suggests these roles are complementary. This supports WHO-recommended integrated care models that blend specialist input with community-based delivery to enhance access in resource-constrained settings.^1^

### Service utilisation based on age

Specialist visit patterns followed a U-shaped curve, with higher utilisation in early childhood, a decline in mid-childhood and a rise again in adolescence. Younger children may receive more services due to heightened parental motivation or perceived urgency. The later increase could reflect the resurgence of therapy needs during adolescence, such as for vocational or social functioning.

Older children’s reliance on CRWs may stem from logistical barriers—for example, school schedules or distance to centres—which make home-based care more viable. These observations, although not directly assessed, align with literature supporting hybrid models that balance intensive early services with long-term community-based support.^26 27^

Considering that the study looks at demographic data from 2015 to 2024, it is possible that certain programme changes might have impacted service utilisation. For example, there was a brief disruption in service provision during the initial days of the COVID-19 pandemic. The programme had to pivot to being entirely tele-rehabilitation based service delivery and is detailed in another publication.^28^ Families with limited access to the internet might have had to drop off or reduce service utilisation in this period. Such changes could impact age-based service utilisation trends and should be kept in mind while interpreting the results. Stakeholder consultation with service providers and parents of children in the programme that led to programmatic changes and increased therapy utilisation is also described in another publication.^17^

### Service utilisation based on sex

The higher male enrolment and utilisation reflect both biological and sociocultural trends. Globally, boys are more frequently diagnosed with NDDs and may exhibit more severe functional impairments.^29 30^ Males are also more likely to display externalising behaviours, which may facilitate earlier recognition and referral.^31 32^

In rural South India, patriarchal norms may drive preferential care for male children, further explaining the observed sex disparities.^33 34^ Although clinical severity could not be directly examined in this dataset, the elevated service use among males may reflect both greater need and inequities in access. These findings emphasise the importance of promoting equitable identification and care for girls, who may exhibit less overt symptoms and risk being underserved.

### Family income and education

Low maternal education and income were significantly associated with reduced service utilisation. Children whose mothers had at least secondary education were substantially more likely to meet the adequate utilisation threshold, supporting global evidence linking maternal education with improved child health outcomes and care engagement.^35 36^

Our findings reinforce the role of caregiver education and household financial stability as critical enablers of consistent rehabilitation access. Targeted outreach, health literacy initiatives and financial support mechanisms could mitigate these barriers and improve service equity. It is important to note that parent mental health status is a factor that can significantly impact therapy service utilisation but was not measured in the present study.

### Policy implications

Our results highlight the unique challenges and patterns of rural South India. They complement and can more specifically inform India’s evolving disability and early intervention policy landscape. Initiatives like Tamil Nadu’s RIGHTS project and the nationwide RBSK screening programme and District Early Intervention Centre therapy programme demonstrate promising steps toward scaling community-based rehabilitation models.^12 37^ The EI programme, with its blended specialist-CRW approach and digital tracking tools, offers a scalable context-specific model that aligns with WHO and national policy priorities for inclusive, accessible and tech-enabled care.

## Limitations and Future Directions

This study was based on retrospective programme data originally designed for service delivery and operational monitoring rather than research. As a result, certain variables were not consistently available across the entire study period, particularly during the early years of implementation. Differences in documentation formats and incomplete discipline-specific therapy records between 2015 and 2018 necessitated the use of a combined service utilisation measure, limiting the ability to examine utilisation patterns by specific therapy type.

The observational design precludes causal inference, and findings reflect associations rather than direct effects. Additionally, the dataset represents a single NGO-led programme operating in three rural districts of South India; therefore, results may not be fully generalisable to other regions, service models or urban contexts. External disruptions, including the temporary shift to tele-rehabilitation during the COVID-19 pandemic, may also have influenced utilisation patterns and should be considered when interpreting age- and diagnosis-related trends. The EI model incorporates scheduled specialist oversight, including monthly follow-up sessions and formal reassessment at least once every 6 months, conducted either in person or via teleconsultation. However, specialists are not continuously embedded within all rural service areas. As a result, the day-to-day implementation is primarily led by CRWs, with specialist input provided at defined intervals and documented through the digital platform. While this hybrid structure is appropriate for geographically dispersed and resource-constrained rural settings, variability in internet connectivity may affect the consistency of specialist oversight between scheduled review intervals.

Future research should employ prospective longitudinal designs with standardised outcome measures to examine how service intensity and modality relate to child functional outcomes over time. Incorporating discipline-specific utilisation data, caregiver mental health indicators and qualitative methods would provide deeper insight into family-level, cultural and systemic factors influencing engagement in intervention. Such approaches would strengthen causal understanding and enhance the policy relevance of large-scale community-based early intervention models.

## Conclusion

This study provides valuable insights into the prevalence, demographic distribution and service utilisation patterns of children with NDDs in rural South India, based on data from Amar Seva Sangam’s EI programme. Findings indicate that younger children benefited from more specialist-led rehabilitation, while older children received greater support from CRWs, reflecting an age-structured, tiered approach to service delivery.

Notably, higher service utilisation was significantly associated with being male, having a mother with at least a secondary school education and belonging to a higher-income household. These findings underscore the importance of addressing gender and socioeconomic disparities in rehabilitation access in South India. While male predominance may reflect both biological vulnerability and sociocultural preferences, the strong influence of maternal education and household income highlights the need for targeted interventions that empower caregivers and reduce structural barriers to care.

The EI programme demonstrates a scalable, community-integrated and technology-enabled model of care that can be customised taking into consideration the findings of this paper, in order to be effectively implemented in resource-limited settings. Expanding such models through policy integration, digital health strategies and community-based engagement holds significant promise for improving long-term developmental outcomes for children with NDDs across India and similar global contexts.

## Supplementary material

10.1136/bmjpo-2025-003663 online supplemental file 1

## Data Availability

Data are available upon reasonable request.
