# Supplementary material for: Understanding barriers to rehabilitation: child and family determinants of service utilisation in the Enabling Inclusion programme in rural South India
Source: BMJ Paediatr Open. 2026 Mar 31;10(1):e003663. doi: 10.1136/bmjpo-2025-003663 (PMC13052689; doi:10.1136/bmjpo-2025-003663)
Supplement: online supplemental file 1 [file bmjpo-10-1-s001.docx]

**Supplemental Table 1. Demographic Information of Children Enrolled in the EI Program® per age of child**

| **Age (Years)** | **Female (%,n)** | **Male (%,n)** | **Total (n)** | **Female Total Days in EI** | **Female Avg. Days in EI** | **Male Total Days in EI** | **Male Avg. Days in EI** |
| --- | --- | --- | --- | --- | --- | --- | --- |
| 0-1 | 51.4% (n=19) | 48.6% (n=18) | 37 | 6,856 | 360.8 | 6,060 | 336.7 |
| 1-2 | 42.1% (n= 24) | 57.9% (n=33) | 57 | 7,288 | 303.7 | 9,413 | 285.2 |
| 2-3 | 39.6% (n=55) | 60.4% (n=84) | 139 | 22,593 | 410.8 | 31,464 | 374.6 |
| 3-4 | 37.1% (n=115) | 62.9% (n=195) | 310 | 73,081 | 635.5 | 106,946 | 548.4 |
| 4-5 | 37.1% (n=180) | 62.9% (n=305) | 485 | 140,977 | 783.2 | 200,971 | 658.9 |
| 5-6 | 38.6% (n=225) | 61.4% (n=358) | 583 | 191,851 | 852.7 | 296,310 | 827.7 |
| 6-7 | 38.8% (n=228) | 61.2% (n=360) | 588 | 193,156 | 847.2 | 343,520 | 954.2 |
| 7-8 | 37.4% (n=230) | 62.6% (n=385) | 615 | 233,780 | 1016.4 | 374,470 | 972.6 |
| 8-9 | 35.4% (n=215) | 64.6% (n=392) | 607 | 229,596 | 1067.9 | 389,874 | 994.6 |
| 9-10 | 36.7% (n=220) | 63.3% (n=380) | 600 | 150,187 | 682.7 | 438,989 | 1155.2 |
| 10-11 | 34.1% (n=170) | 65.9% (n=328) | 498 | 200,475 | 1179.3 | 355,261 | 1083.1 |
| 11-12 | 48.6% (n=203) | 51.4% (n=215) | 418 | 217,911 | 1073.5 | 263,486 | 1225.5 |
| 12-13 | 50.3% (n=167) | 49.7% (n=165) | 332 | 138,147 | 827.2 | 234,944 | 1423.9 |
| 13-14 | 54.2% (n=130) | 45.8% (n=110) | 240 | 90,567 | 696.7 | 161,308 | 1466.4 |
| 14-15 | 58.6% (n=85) | 41.4% (n=60) | 145 | 45,674 | 537.3 | 61,304 | 1021.7 |
| 15-16 | 69.7% (n=99) | 30.3% (n=43) | 142 | 47,100 | 475.8 | 59,522 | 1384.2 |
| 16-17 | 67.0% (n=75) | 33.0% (n=37) | 112 | 38,040 | 507.2 | 45,972 | 1242.5 |
| 17-18 | 64.5% (n=60) | 35.5% (n=33) | 93 | 31,213 | 520.2 | 47,245 | 1431.7 |
| 18-19 | 61.8% (n=42) | 38.2% (n=26) | 68 | 30,460 | 725.2 | 26,358 | 1013.8 |
| Total | 41.9% (n=2542) | 58.1% (n=3527) | 6069 | 2,088,952 | 822.2 | 3,453,417 | 978.9 |

**Supplemental Table 2: Specialist and CRW Visits by Age and Sex while enrolled in the program**

| **Age (Years)** | **Sex** | **Specialist Visits (Min-Max)** | **Specialist Average Visit** | **Specialist SD** | **CRW Visits (Min-Max)** | **CRW Average Visit** | **CRW SD** |
| --- | --- | --- | --- | --- | --- | --- | --- |
| 0-1 | Female | 1174 (5-62) | 61.8 | 14.25 | 1639 (11-75) | 86.3 | 16 |
| 0-1 | Male | 965 (4-72) | 53.6 | 17 | 1687 (9-80) | 93.7 | 17.75 |
| 1-2 | Female | 1413 (8-183) | 58.9 | 43.75 | 1915 (52-175) | 79.8 | 30.75 |
| 1-2 | Male | 998 (35-143) | 30.2 | 27 | 1628 (45-210) | 49.3 | 41.25 |
| 2-3 | Female | 2453 (2-353) | 44.6 | 87.75 | 3298 (17-265) | 60.0 | 62 |
| 2-3 | Male | 2300 (1-293) | 27.4 | 73 | 3051 (2-342) | 36.3 | 85 |
| 3-4 | Female | 3626 (21-450) | 30.5 | 107.25 | 4339 (19-368) | 36.5 | 87.25 |
| 3-4 | Male | 5998 (15-419) | 29.4 | 101 | 6970 (44-446) | 34.2 | 100.5 |
| 4-5 | Female | 5663 (1-462) | 30.6 | 115.25 | 7092 (2-415) | 38.3 | 103.25 |
| 4-5 | Male | 13369 (15-582) | 43.1 | 141.75 | 16490 (4-487) | 53.2 | 121.75 |
| 5-6 | Female | 6835 (4-519) | 30.4 | 128.75 | 9083 (1-397) | 40.4 | 99 |
| 5-6 | Male | 13106 (3-522) | 36.6 | 129.75 | 15491 (1-430) | 43.3 | 107.25 |
| 6-7 | Female | 6660 (1-634) | 29.2 | 158.25 | 8551 (21-514) | 37.5 | 123.25 |
| 6-7 | Male | 12471 (1-487) | 32.8 | 121.5 | 12914 (1-429) | 34.0 | 107 |
| 7-8 | Female | 5858 (12-584) | 24.4 | 143 | 9307 (1-487) | 38.8 | 123.25 |
| 7-8 | Male | 9644 (1-676) | 24.1 | 168.75 | 12344 (1-392) | 30.9 | 107 |
| 8-9 | Female | 6246 (5-548) | 28.3 | 135.75 | 7647 (9-517) | 34.6 | 121.5 |
| 8-9 | Male | 11766 (1-775) | 30.0 | 193.5 | 13274 (1-460) | 33.9 | 97.75 |
| 9-10 | Female | 8500 (1-666) | 35.6 | 166.25 | 8703 (1-355) | 36.4 | 88.75 |
| 9-10 | Male | 14200 (1-871) | 35.7 | 217.5 | 13393 (1-477) | 33.7 | 119.25 |
| 10-11 | Female | 5524 (8-648) | 29.9 | 160 | 5371 (4-565) | 29.0 | 140.25 |
| 10-11 | Male | 10787 (2-530) | 32.9 | 132 | 13833 (2-482) | 42.2 | 120 |
| 11-12 | Female | 7586 (2-515) | 36.1 | 128.25 | 9040 (1-375) | 43.1 | 93.5 |
| 11-12 | Male | 6407 (2-794) | 27.7 | 198 | 7223 (11-439) | 31.3 | 107 |
| 12-13 | Female | 3559 (1-497) | 21.3 | 124 | 3783 (4-452) | 22.7 | 112 |
| 12-13 | Male | 10636 (1-605) | 64.5 | 151 | 10420 (9-371) | 63.2 | 149 |
| 13-14 | Female | 4895 (6-705) | 36.3 | 174.75 | 5397 (1-482) | 40.0 | 120.25 |
| 13-14 | Male | 5004 (6-535) | 42.1 | 132.25 | 7653 (6-438) | 64.3 | 108 |
| 14-15 | Female | 7723 (2-648) | 85.8 | 161.5 | 8331 (2-298) | 92.6 | 74 |
| 14-15 | Male | 6768 (1-807) | 98.1 | 201.5 | 7974 (9-350) | 115.6 | 99.5 |
| 15-16 | Female | 6250 (3-689) | 72.4 | 150.5 | 7050 (2-315) | 80.3 | 82.5 |
| 15-16 | Male | 6893 (2-750) | 90.2 | 180.25 | 8124 (4-365) | 103.7 | 97.2 |
| 16-17 | Female | 5802 (1-678) | 67.1 | 142.75 | 6701 (1-290) | 75.5 | 76.8 |
| 16-17 | Male | 7155 (3-821) | 95.3 | 190.1 | 8309 (5-380) | 108.2 | 99.6 |
| 17-18 | Female | 5420 (2-645) | 62.8 | 130.5 | 6358 (3-275) | 71.2 | 71.3 |
| 17-18 | Male | 7392 (1-890) | 100.5 | 200.4 | 8497 (7-390) | 112.4 | 103 |
