# Supplementary material for: Understanding barriers to rehabilitation: child and family determinants of service utilisation in the Enabling Inclusion programme in rural South India
Source: BMJ Paediatr Open. 2026 Mar 31;10(1):e003663. doi: 10.1136/bmjpo-2025-003663 (PMC13052689; doi:10.1136/bmjpo-2025-003663)
Supplement: Supplementary file 2 [file bmjpo-2025-003663.reviewer_comments.pdf]

PEER REVIEW HISTORY

BMJ Paediatrics Open publishes all reviews undertaken for accepted manuscripts. Reviewers are asked to complete a checklist review form and are provided with free text boxes to elaborate on their assessment. These free text comments are reproduced below.

ARTICLE DETAILS

**TITLE (PROVISIONAL)**	Understanding Barriers to Rehabilitation: Child and Family Determinants of Service Utilization in the Enabling Inclusion® Program in Rural South India
**AUTHORS**	Coutinho, Franzina<br>Saxena, Gauri<br>Brien, Marie<br>Poojari, Deepalaxmi<br>Ramachandran, Selvam<br>Bhrasadiya, Nisha<br>Srinivasan, Sankara Raman<br>Venkatachalapathy, Navamani<br>Dahan-Oliel, Noemi<br>Krishna, Dinesh

VERSION 1 – REVIEW

**REVIEWER NAME**	Bhojne- Kasar, Usha
**REVIEWER AFFILIATION**	King Edward Memorial Hospital and Seth Gordhandas Sunderdas Medical College, Occupational Therapy
**REVIEWER CONFLICT OF INTEREST**	No Conflict of Interest
**AI DISCLOSURE**	No, I have not used AI
**DATE REVIEW RETURNED**	24-May-2025

**GENERAL COMMENTS**	Including parent or caretakers mental health status as one of the factor for therapy compliance is recommended.

**REVIEWER NAME**	Ramkumar, Vidya
**REVIEWER AFFILIATION**	Sri Ramachandra Institute of Higher Education and Research (Deemed to be University), Sri Ramachandra Faculty of Audiology and Speech Language Pathology
**REVIEWER CONFLICT OF INTEREST**	No Conflict of Interest
**AI DISCLOSURE**	No, I have not used AI
**DATE REVIEW RETURNED**	23-Jul-2025

**GENERAL COMMENTS**	This is an important paper that describes the results of rehabilitation uptake in a rural setting in southern India. It is now well established that institution based models do not serve a large majority of individuals who need rehabilitation.<br><br>While the paper has merits in.....the following are some critical comments for the authors to consider/clarify.<br><br>1. The abstract suggests that speech language disorder to be a

	diagnosis. This is odd. Were they having no other problems? e.g. did they include only persons with stuttering, misarticulation, or a language delay without any underlying diagnosis ?<br><br>2. The introduction is brief and does not adequately highlight the various service options available for NDD in the Indian context. Since both public and private organisations offer them, only mention of DEIC based service limits the understanding of the context. Even though it is understandable that rural population depends largely on public health infrastructure. Similarly the DEIC data must be validated with a suitable citation.<br>The focus shifts too quickly to Amar seva model and gas are claimed purely based on this.<br>It would be important to highlight the efforts of other community efforts in India towards rehabilitation. e.g Mobility India. And then differntuate Amar Seva's model from these.<br>Community-based rehabilitation using technology/ task shifting also exists for hearing and speech disorders in India. These maybe suitably referenced in both introduction and discussion, to both authenticate the gap as well as compare what you found. These will help represent the context more appropriately.<br><br>Even within the Amar Seva model it is not clear what types of rehabilitation services are included? OT/ST/Aural rehabilitation/PT/Special education? it is difficult to evaluate this manuscript without sufficient understanding of how services were delivered. The study would be of interest to readership only when this information is known. There is only a brief description of the Enabling inclusion platform, but some details on the service is important to understanding the barriers/ how it was broken .<br><br>Most importantly the need/ gap assessment is not centred on what is available and missing with respect to understanding service utilisation?<br><br>Aim and Title do not go together. The title is misleading with the phrase "breaking barriers to rehabilitation" when the study is only about reviewing uptake. This can be dropped from the title to represent the study more accurately.<br><br><br>Method<br>If number of sessions attended through the EI program is the measure, then why were children excluded if they attended other therapies before?<br><br>Was the app available from 2015? or were manual records updated in the app? what was the data filing method followed before the app if app was not there since 2015?<br><br>While the quantitative statistical data is useful, it would be important know programmatic changes that may have contributed to uptake. E.g. were all therapists available all the time between 2015 to 2024? if therapists were unavailable for periods of time, it may result in drop outs. How are these factored? these must be discussed.<br><br><br>In the section on diagnosis and time spent, It would be helpful to know which system is being followed, as it is usual to say speech disorders as a diagnosis unless it is isolated condition, in which case

	one must mention it as stuttering / mis articulation. Speech language disorders are usually a symptom in ID, autism, HI, and so on.<br><br>It is intriguing that "therapy" is seen as one single entity throughout the paper. Service utilisation for OT/ ST/ Aural rehab/ PT should be stated separately. As uptake also depends on these types.<br><br>Overall, the paper seems to represent the use of a platform to track patient data/ management in rehabilitation rather than update of a EI program / model.<br><br>The conclusions are strong for a descriptive analysis of recorded data. An implementation study with clear outcomes of benefit using mixed methods designs would be more appropriate to make these conclusions related to scaling, and contextual relevance.

VERSION 1 – AUTHOR RESPONSE

Reviewer: 1
Dr. Usha Bhojne- Kasar, King Edward Memorial Hospital and Seth Gordhandas Sunderdas Medical College, P D Hinduja National Hospital and Medical Research Centre

Comments to the Author
Including parent or caretaker's mental health status as one of the factors for therapy compliance is recommended.

- Caretake mental health status has been included in the discussion as one of the factors that might impact therapy compliance

Reviewer: 2
Dr. Vidya Ramkumar, Sri Ramachandra Institute of Higher Education and Research (Deemed to be University)

Comments to the Author
This is an important paper that describes the results of rehabilitation uptake in a rural setting in southern India. It is now well established that institution based models do not serve a large majority of individuals who need rehabilitation.

While the paper has merits in.....the following are some critical comments for the authors to consider/clarify.

1. The abstract suggests that speech language disorder to be a diagnosis. This is odd. Were they having no other problems? e.g. did they include only persons with stuttering, misarticulation, or a language delay without any underlying diagnosis?

- In our dataset, “Speech Language Disorder” was recorded as a standalone diagnosis in cases where the child’s primary presenting difficulty was related to speech and/or language (e.g., stuttering, articulation delay, expressive/receptive language delay), and where no other neurodevelopmental or intellectual disability diagnosis had been made at the time of enrolment. This reflects the way data was recorded in the EI program intake records rather than formal diagnostic category. Early assessment, in rural contexts, may focus on the most observable presenting difficulty, with a broader underlying diagnosis only emerging later. For the purpose of this analysis, these cases were grouped together as “Speech Language Disorder” to represent children who initially presented without comorbid diagnoses.

2. The introduction is brief and does not adequately highlight the various service options available for NDD in the Indian context. Since both public and private organisations offer them, only mention of DEIC based service limits the understanding of the context. Even though it is understandable that rural population depends largely on public health infrastructure. Similarly the DEIC data must be validated with a suitable citation.
The focus shifts too quickly to Amar seva model and gas are claimed purely based on this.
It would be important to highlight the efforts of other community efforts in India towards rehabilitation. e.g Mobility India. And then differentiate Amar Seva's model from these.
Community-based rehabilitation using technology/ task shifting also exists for hearing and speech disorders in India. These maybe suitably referenced in both introduction and discussion, to both authenticate the gap as well as compare what you found. These will help represent the context more appropriately.

- The introduction has been rewritten to describe the context of rehabilitation efforts in India and highlight the differences between existing work and the work done by ASSA (Page 5-6)

3. Even within the Amar Seva model it is not clear what types of rehabilitation services are included? OT/ST/Aural rehabilitation/PT/Special education? it is difficult to evaluate this manuscript without sufficient understanding of how services were delivered. The study would be of interest to readership only when this information is known. There is only a brief description of the Enabling inclusion platform, but some details on the service is important to understanding the barriers/ how it was broken
.

- The Amar Seva EI model includes Occupational Therapy (OT), Speech Therapy (ST), Physiotherapy (PT), Special Education, and Aural Rehabilitation. In the dataset used for this study, service utilisation was calculated using the total number of therapy sessions attended across all rehabilitation types. Due to differences in early documentation formats, individual counts for each therapy type were not consistently available in the program's historical datasets (2015–2018). While recent EI records capture discipline-specific data, combining all therapies into a single utilisation measure ensured comparability across the full 2015–2024 dataset.

4. Most importantly the need/ gap assessment is not centred on what is available and missing with respect to understanding service utilisation?

- A sentence on the need for understanding service utilization has been added on Page 6

5. Aim and Title do not go together. The title is misleading with the phrase "breaking barriers to rehabilitation" when the study is only about reviewing uptake. This can be dropped from the title to represent the study more accurately.

- The title has been edited to **"Understanding Barriers to Rehabilitation: Child and Family Determinants of Service Utilization in the Enabling Inclusion® Program in Rural South India"** to better reflect the aims of the study

Method
6. If the number of sessions attended through the EI program is the measure, then why were children excluded if they attended other therapies before?

- We excluded children with prior therapy history outside the EI program to reduce confounding for variables such as initial skill level, rate of progress, and utilisation patterns, which would make it harder to isolate the relationship between child/family factors and utilisation within the EI program itself. This ensured that since all participants started with the EI program as their primary structured intervention, making comparisons within the cohort became more internally valid. The manuscript has been edited to reflect this.

7. Was the app available from 2015? or were manual records updated in the app? what was the data filing method followed before the app if app was not there since 2015?

- The app has been available since 2015 so there has been consistency in data filing.

8. While the quantitative statistical data is useful, it would be important know programmatic changes that may have contributed to uptake. E.g. were all therapists available all the time between 2015 to 2024? if therapists were unavailable for periods of time, it may result in drop outs. How are these factored? these must be discussed.

- A section on programmatic changes has been included in the discussion on Page 19.
- Programmatic changes and impact on uptake were also described in 2 other papers: Krishna, D., Mutthukarrupan, S., Bharathwaj, A., Ponnusamy, R., Srinivasan, S., et al. Rapid-cycle evaluation in an early intervention program for children in South India: optimizing service providers' quality of work-life, family program engagement and school enrollment. Frontiers in Public Health. (2020), as well as, Brien, M., Coutinho, F., Krishna, D., van der Haar, L., de Laat, J., Srinivasan, S. R., & Venkatachalapathy, N. (2023). Leveraging monitoring, evaluation, and learning to scale the Enabling Inclusion® program for children with disabilities in India and globally. *Frontiers in public health*, *11*, 1165034. https://doi.org/10.3389/fpubh.2023.1165034

9. In the section on diagnosis and time spent, It would be helpful to know which system is being followed, as it is usual to say speech disorders as a diagnosis unless it is isolated condition, in which case one must mention it as stuttering / mis articulation. Speech language disorders are usually a symptom in ID, autism, HI, and so on.

- Throughout the manuscript "speech language disorders" have been used as a stand alone diagnosis as long as no other NDD was present. The manuscript has been edited to make this clear.

10. It is intriguing that "therapy" is seen as one single entity throughout the paper. Service utilisation for OT/ ST/ Aural rehab/ PT should be stated separately. As uptake also depends on these types.

- The Amar Seva EI model includes Occupational Therapy (OT), Speech Therapy (ST), Physiotherapy (PT), Special Education, and Aural Rehabilitation. In the dataset analysed, service utilisation has been calculated using the total number of therapy sessions attended across all rehabilitation types. This is because separate counts for each therapy type were not consistently available in the program's historical datasets from 2015–2018 due to differences in early documentation formats. While recent EI app records capture discipline-specific data, combining all therapies into a single utilisation measure ensured comparability across the full 2015–2024 dataset. Future research will focus on the differences between these services.

Overall, the paper seems to represent the use of a platform to track patient data/ management in rehabilitation rather than update of a EI program / model.

The conclusions are strong for a descriptive analysis of recorded data. An implementation study with clear outcomes of benefit using mixed methods designs would be more appropriate to make these conclusions related to scaling, and contextual relevance.

VERSION 2 – REVIEW

**REVIEWER NAME**	Ramkumar, Vidya
**REVIEWER AFFILIATION**	Sri Ramachandra Institute of Higher Education and Research (Deemed to be University), Sri Ramachandra Faculty of Audiology and Speech Language Pathology

REVIEWER CONFLICT OF INTEREST	No Conflict of Interest
AI DISCLOSURE	No, I have not used AI
DATE REVIEW RETURNED	09-Oct-2025

GENERAL COMMENTS	The authors may revise and resubmit the manuscript after addressing all the suggestions in a more comprehensive manner .<br><br>A critical feedback raised on the lack of rigor in needs/ gap assessment has not been addressed (a one sentence change is indicated).<br><br>Overall the manuscript requires better capturing of on-going efforts as a simple google scholar search yields results on these (even those included do not have citations) greater descriptions of the model, the operational definitions of therapies, speech language disorder as diagnosis, and limitations in data set availability to mention a few.

VERSION 2 – AUTHOR RESPONSE

Reviewer: 2

Dr. Vidya Ramkumar, Sri Ramachandra Institute of Higher Education and Research (Deemed to be University)

Comments to the Author

The authors may revise and resubmit the manuscript after addressing all the suggestions in a more comprehensive manner .

1. A critical feedback raised on the lack of rigor in needs/ gap assessment has not been addressed (a one sentence change is indicated).
    - The introduction has been entirely rewritten to better highlight the gap assessment for this study.
    - Additions have been made to the Discussion on pages 22, 24, and 27

2. Overall the manuscript requires better capturing of on-going efforts as a simple google scholar search yields results on these (even those included do not have citations) greater descriptions of the model, the operational definitions of therapies, speech language disorder as diagnosis, and limitations in data set availability to mention a few.
    - Descriptions of the models have been added on Page 7
    - Operational definitions and limitations in data set availability have been added on Page 7, 11 and 12
    - A section explaining the rationale for ‘speech language disorder’ as a diagnosis has been added on Page 13

- The limitations section of the manuscript has been entirely rewritten (Pages 27-28)

VERSION 3 – REVIEW

**REVIEWER NAME**	Bhojne- Kasar, Usha
**REVIEWER AFFILIATION**	King Edward Memorial Hospital and Seth Gordhandas Sunderdas Medical College, Occupational Therapy
**REVIEWER CONFLICT OF INTEREST**	No Conflict of Interest
**AI DISCLOSURE**	No, I have not used AI
**DATE REVIEW RETURNED**	01-Jan-2026

**GENERAL COMMENTS**	Paper is accepted

**REVIEWER NAME**	Ramkumar, Vidya
**REVIEWER AFFILIATION**	Sri Ramachandra Institute of Higher Education and Research (Deemed to be University), Sri Ramachandra Faculty of Audiology and Speech Language Pathology
**REVIEWER CONFLICT OF INTEREST**	No Conflict of Interest
**AI DISCLOSURE**	No, I have not used AI
**DATE REVIEW RETURNED**	14-Jan-2026

**GENERAL COMMENTS**	The authors have addressed the comments to a reasonable extent. But the limitations really highlights the shortcomings in this data set. The results of this paper must be read in the context of the limitations for a careful interpretation.<br>The first paragraph of limitations can be removed as it does not highlight limitation or future direction

VERSION 3 – AUTHOR RESPONSE

Reviewer: 1

Dr. Usha Bhojne- Kasar, King Edward Memorial Hospital and Seth Gordhandas Sunderdas Medical College, P D Hinduja National Hospital and Medical Research Centre

Comments to the Author

Paper is accepted

Reviewer: 2

Dr. Vidya Ramkumar, Sri Ramachandra Institute of Higher Education and Research (Deemed to be University)

Comments to the Author

Reviewer: 2

Dr. Vidya Ramkumar, Sri Ramachandra Institute of Higher Education and Research (Deemed to be University)

Comments to the Author

The authors have addressed the comments to a reasonable extent. But the limitations really highlight the shortcomings in this data set. The results of this paper must be read in the context of the limitations for a careful interpretation.

The first paragraph of limitations can be removed as it does not highlight limitation or future direction

- Additions have been made to the discussion on Pages 18, 20, 21 and 23 to reflect the limitations of the dataset and to aid in interpretations of the results in the context of said limitations.
- The limitations section has been rewritten to remove unnecessary information and include more context on Page 23 of the manuscript.

VERSION 4 – REVIEW

**REVIEWER NAME**	Bhojne- Kasar, Usha
**REVIEWER AFFILIATION**	King Edward Memorial Hospital and Seth Gordhandas Sunderdas Medical College, Occupational Therapy
**REVIEWER CONFLICT OF INTEREST**	No Conflict of Interest
**AI DISCLOSURE**	No, I have not used AI
**DATE REVIEW RETURNED**	02-Feb-2026

**GENERAL COMMENTS**	Paper is accepted

**REVIEWER NAME**	Ramkumar, Vidya
**REVIEWER AFFILIATION**	Sri Ramachandra Institute of Higher Education and Research (Deemed to be University), Sri Ramachandra Faculty of Audiology and Speech Language Pathology
**REVIEWER CONFLICT OF INTEREST**	No Conflict of Interest
**AI DISCLOSURE**	No, I have not used AI
**DATE REVIEW RETURNED**	11-Feb-2026

**GENERAL COMMENTS**	The revised version maybe accepted
